# Fibrinolytics and Intraventricular Hemorrhage: A Systematic Review and Meta-analysis

**DOI:** 10.1007/s12028-019-00786-5

**Published:** 2019-08-02

**Authors:** Thomas S. van Solinge, Ivo S. Muskens, Vasileios K. Kavouridis, William B. Gormley, Rania A. Mekary, Marike L. D. Broekman, Omar Arnaout

**Affiliations:** 1 Computational Neurosciences Outcome Center, Brigham and Women’s Hospital, Harvard Medical School, Boston, MA USA; 2 Department of Neurology, Massachusetts General Hospital, Harvard Medical School, Boston, MA USA; 3 Department of Neurosurgery, Leiden University Medical Center, Albinusdreef 2, 2333 ZA Leiden, The Netherlands; 4 Department of Pharmaceutical Business and Administrative Sciences, School of Pharmacy, MCPHS University, Boston, MA USA; 5 Department of Neurosurgery, Haaglanden Medical Center, The Hague, The Netherlands

**Keywords:** Cerebral ventricles, Cerebral hemorrhage, External ventricular drain, Intraventricular, Fibrinolytic agents

## Abstract

Intraventricular hemorrhage (IVH) is an independent poor prognostic factor in subarachnoid and intra-parenchymal hemorrhage. The use of intraventricular fibrinolytics (IVF) has long been debated, and its exact effects on outcomes are unknown. A systematic review and meta-analysis were performed in accordance with the PRISMA guidelines to assess the impact of IVF after non-traumatic IVH on mortality, functional outcome, intracranial bleeding, ventriculitis, time until clearance of third and fourth ventricles, obstruction of external ventricular drains (EVD), and shunt dependency. Nineteen studies were included in the meta-analysis, totaling 1020 patients. IVF was associated with lower mortality (relative risk [RR] 0.58; 95% confidence interval [CI] 0.47–0.72), fewer EVD obstructions (RR 0.41; 95% CI 0.22–0.74), and a shorter time until clearance of the ventricles (median difference [MD] − 4.05 days; 95% CI − 5.52 to − 2.57). There was no difference in good functional outcome, RR 1.41 (95% CI 0.98–2.03), or shunt dependency, RR 0.93 (95% CI 0.70–1.22). Correction for publication bias predicted an increased risk of intracranial bleeding, RR 1.67 (95% CI 1.01–2.74) and a lower risk of ventriculitis, RR 0.68 (95% CI 0.45–1.03) in IVH patients treated with IVF. IVF was associated with improved survival, faster clearance of blood from the ventricles and fewer drain obstructions, but further research is warranted to elucidate the effects on ventriculitis, long-term functional outcomes, and re-hemorrhage.

**Electronic supplementary material:**

The online version of this article (10.1007/s12028-019-00786-5) contains supplementary material, which is available to authorized users.

## Introduction

The presence of an intraventricular hemorrhage (IVH) in subarachnoid hemorrhage (SAH), intra-parenchymal hemorrhage (IPH), and, to a lesser extent, traumatic brain injury is an independent risk factor for poor outcomes [1, 2]. By obstructing the flow of cerebrospinal fluid (CSF), IVH can cause acute hydrocephalus necessitating CSF diversion, typically in the form of an external ventricular drain (EVD) [1]. Management of this EVD is often complicated by obstruction of the shunt with blood clots, which leads to poor intracranial pressure control and an increased risk of infection [3]. Multiple replacements of EVDs and long EVD drainage are regularly needed when managing post-IVH hydrocephalus, which are both linked to an increased risk of ventriculitis [4, 5]. Aside from hydrocephalus, the mass effect of the blood clot can hamper perfusion of local tissue leading to ischemia [6], while the presence of blood and blood-degradation products in the CSF contributes to periventricular edema, neural cell death, and arachnoidal fibrosis [7]. Combined, these factors conduce to the development of communicating hydrocephalus, with many patients showing persistent dependence on CSF diversion long after the dissolvement of the initial blood clot, necessitating placement of permanent shunts [8, 9].

To address the complications related to IVH and IVH-related EVD use, efforts have been made to accelerate the removal of ventricular blood. The intraventricular injection of fibrinolytic agents, referred to as intraventricular fibrinolysis (IVF), in patients suffering from IVH was first described in 1990 [10] and has thus far shown mixed results [11–14]. The American Heart Association/American Stroke Association guidelines conclude that the efficacy and safety of fibrinolytics in IVH are uncertain [15]. Recently, the CLEAR-III study [14] evaluated the effect of IVF for IVH resulting from small IPHs and observed a decreased risk of mortality, which was endorsed by previous meta-analyses [16, 17], while no improvement of good functional outcome (GFO) (modified Rankin Score [mRS] equal to or lower than 3) was observed [14]. Previous systematic reviews have reached different conclusions regarding functional outcome, and discussions regarding the safety of (repeated) injections of fibrinolytic agents into fresh hemorrhages are ongoing [16–19]. Moreover, a thorough meta-analysis including meta-regression and assessment of publication bias in different outcomes has thus far lacked.

In light of the conflicting results in literature and the recent findings of the CLEAR-III trial [14], we sought to provide an updated systematic review and meta-analysis regarding the use of fibrinolytics in the treatment of non-traumatic IVH. We assessed patient mortality, functional outcome, shunt dependency and time until clearance of the ventricles, as well as complications related to EVD treatment including obstruction rate, ventriculitis, and incidence of post-treatment intracerebral hemorrhage.

## Methods

### Literature Search

This systematic review and meta-analysis was conducted in accordance with the Preferred Reporting Items for Systematic Reviews and Meta-Analyses guidelines [20]. All data were gathered from publicly available sources. In December of 2017, we searched the PubMed and Embase databases for articles comparing the use of intraventricular fibrinolytics via EVD versus EVD alone in the treatment of non-traumatic IVH. Keywords, MeSH terms, and Emtree terms including IVH, cerebral hemorrhage, clot, thrombus, thrombolysis, fibrinolytic therapy, plasminogen activator, and synonyms were combined with the help of a librarian to form our search strategy (Supplementary Table I). Both the title/abstract screening and the full-text screening were performed independently by two reviewers (T.S. and V.K.), with discrepancies resolved via discussion with a third reviewer (O.A.). The reference lists of full-text articles were screened for additional studies.

### Study Selection

Studies were excluded if they were not written in the English language or if the full text was not available. When the outcomes of studies were reported in multiple publications, the publication with the largest patient cohort with relevant outcome data was included in this review. Results from case reports, reviews, registry data, abstracts, and replies/commentaries were excluded. Eligibility of studies was assessed using the following PICOS criteria: Participants (P): patients with a non-traumatic IVH; Intervention (I): EVD with injection of fibrinolytics; Control (C): EVD alone; Outcomes (O): mortality, functional outcome, occurrence of ventriculitis, shunt dependency, intracranial bleeding after start of treatment, obstruction of EVD, and time until clearance of the third and fourth ventricles; and Study design (S): we selected randomized controlled trials (RCTs), prospective cohort studies and, due to the scarcity of studies, also included retrospective cohort studies and matched case–control series. For inclusion criteria and details regarding data extraction, see Supplementary Methods.

### Data Extraction

The following data were extracted from each study: name of first author, year of publication, journal of publication, country of origin, trial design, inclusion and exclusion criteria, treatment, number of patients, time of follow-up, end-point mortality, functional outcomes and incidences of ventriculitis, shunt placement, symptomatic bleeding, obstruction rates, and time until clearance of third and fourth ventricles on computed tomography (CT). GFO was defined as a score of 3 or lower on the mRS or 4 and higher on the Glasgow Outcome Scale (GOS), assessed at least 3 months after start of therapy. ‘Intracranial bleeding’ was defined as symptomatic hemorrhage after the start of intraventricular treatment. For patient characteristics, mean age and sex were extracted. The quality of non-randomized studies was assessed using the Newcastle–Ottawa scale (NOS) for non-randomized studies [21] and the Jadad-score [22] for RCTs. Assessment was done by two reviewers (T.S. and V.K.), with discrepancies solved through discussion. A study was considered of higher quality if the score was equal to or higher than the median score of the studies included (4 > = for Jadad, 6 > = for NOS).

### Statistical Analysis

For outcome analyses, both random- and fixed-effect models were used to obtain risk ratios (RRs) with 95% confidence intervals (CIs) for dichotomous outcomes. Forest plots were created using the random-effects model. For continuous outcomes, we extracted the reported means and evaluated the mean difference using both the random- and fixed-effect models, with the forest plot created with the random-effects model. Heterogeneity was assessed using Cochran’s *Q* test (*p* < 0.10) and the *I*^2^ statistic. If *I*^2^ > 50%, heterogeneity was deemed considerable [23]. Details considering the meta-regression analysis and assessment of publication bias via the trim-fill method can be found in Supplementary Methods. *p* values < 0.05 were considered statistically significant unless otherwise specified. All analyses were performed in R v3.4.1 (The R Foundation for Statistical Computing) using the ‘Metafor’ package [24].

## Results

Our search strategy resulted in the retrieval of 3518 unique studies. After careful screening and assessment, nineteen articles were included in the meta-analysis (Supplementary Fig. 1) [11–14, 25–39]. Eight studies were classified as RCTs [14, 25, 29, 30, 33, 37–39], six as retrospective cohort studies [11, 12, 28, 31, 34, 35], and five as matched case–control studies [13, 26, 27, 32, 36]. The majority of studies focused on IVH in the setting of IPH (68%) compared to SAH (26%), with the remainder including both IPH and SAH patients (5%). The use of recombinant tissue plasminogen activator ((r)t-PA) increased over the years and was slightly more common than urokinase, 55% versus 45% of the studies, respectively. Additional study details can be found in Table 1.

**Table 1:** Study characteristics

References	Region	Design	Origin of IVH	Patients		Fibrinolytic	Dose	Quality (NOS/Jadad)	Impact factor
				IVF + EVD	EVD				
Akdemir et al. [30]	Middle East	RCT	IPH and SAH	7	9	UK	5000 IU/12 h	2^a^	2.06
Coplin et al. [34]	N-America	RCS	IPH	22	18	UK	10,000 IU/12 h	6^b^	5.72
Ducruet et al. [35]	N-America	RCS	IPH	13	17	tPA	1–3 mg/12 h	5^b^	4.89
Dunatov et al. [12]	Europe	RCS	IPH	48	49	rt-PA	1 mg/12 h	5^b^	3.09
Findlay et al. [31]	N-America	RCS	SAH	21	9	rt-PA	4 mg/24 h	5^b^	4.89
Gerner et al. [32]	Europe	M-CS	SAH	14	14	rt-PA	1 mg/8 h	7^b^	2.75
Hallevi et al. [11]	Middle East	RCS	IPH	18	11	tPA	1–2 mg/24 h	5^b^	2.47
Hanley et al. [14]	N-America	RCT	IPH	249	251	tPA	1 mg/8 h	5^a^	44
Huttner et al. [36]	Europe	M-CS	IPH	22	22	rt-PA	2–4 mg/12 h	8^b^	3.96
King et al. [37]	Asia	RCT	IPH	7	9	UK	25,000 IU/12 h	5^a^	1.38
Kramer et al. [33]	N-America	RCT	SAH	6	6	tPA	2 mg/12 h	5^a^	2.75
Litrico et al. [38]	Europe	RCT	SAH	11	8	rt-PA	3 mg/12 h	3^a^	2.06
Naff et al. [39]	N-America	RCT	IPH	6	5	UK	25,000 IU/12 h	4^a^	4.89
Naff et al. [25]	N-America	RCT	IPH	26	22	rt-PA	3 mg/12 h	3^a^	5.72
Rainov and Burkert [26]	Europe	M-CS	IPH	16	5	UK	10,000 IU/12 h	6^b^	2.06
Todo et al. [27]	Asia	CS	IPH and SAH	6	4	UK	10,000 IU/12 h	4^b^	3.74
Torres et al. [28]	Europe	RCS	IPH	14	14	UK	10,000 IU/12 h	6^b^	0.96
Tung et al. [29]	Asia	RCT	IPH	10	11	UK	50,000/12 h	1^a^	0.96
Varelas et al. [13]	N-America	M-CS	SAH	10	10	tPA	2 mg/12 h	7^b^	4.89

*CS* case–control, *EVD* extraventricular drain, *IPH* intra-parenchymal hemorrhage, *IU* international units, *IVH* intraventricular hemorrhage, *IVF* intraventricular fibrinolysis, *M*-*CS* matched case–control, *mg* milligram, *NA* not assessed, *NOS* Newcastle–Ottawa Outcome Scale, *RCS* retrospective cohort study, *RCT* randomized controlled trial, *rt*-*PA* recombinant tissue plasminogen activator, *SAH* subarachnoid hemorrhage, *tPA* tissue plasminogen activator, *UK* urokinase

^a^Scored using Jadad scale, 1–5 points

^b^Scored using NOS scale, 1–9 points (high quality defined as a score of 4 > = for Jadad, 6 > = for NOS)

Collectively, 1020 patients were included in this meta-analysis of which 526 received intraventricular fibrinolytics. Male patients constituted 55.9% of the studied population, and the overall mean age was 56 years (median 56 years). Patients who received IVF had a mean age of 56.0 years (median 55.5), while those receiving EVD only had a mean age of 56.1 years (median 56). More study details and the assessment of bias regarding RCTs can be found in Table I, Supplementary Tables 2–5, and in Supplementary Results section.

### Mortality

**Fig. 1:**
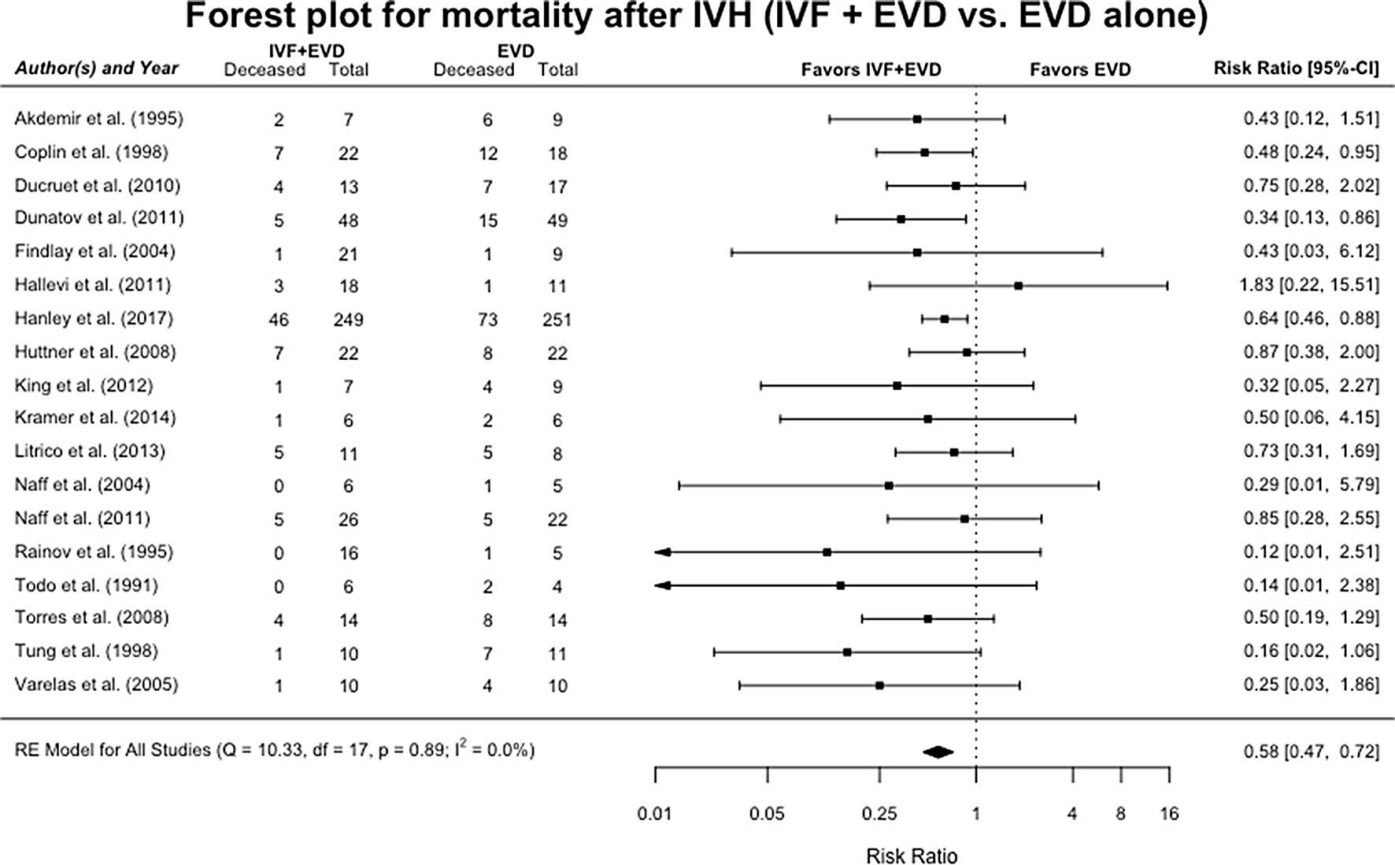
Forest plot for mortality after intraventricular hemorrhage. Pooled risk ratios for mortality in patients receiving IVF and EVD versus those being treated with EVD alone, in a random-effects model. Solid squares represent the point estimate of each study, with 95% CI being shown in error bars. The diamond represents the pooled estimate of the risk ratios. *I*^2^ and *p* values for heterogeneity are shown. *CI* confidence interval, *EVD* external ventricular drain, *IVF* intraventricular fibrinolysis, *IVH* intraventricular hemorrhage, *RE* random effects

Eighteen studies reported on mortality in patients with IVH [11–14, 25–31, 33–39]. Pooled analysis showed a significant decrease in mortality risk for patients receiving IVF treatment with EVD compared to patients receiving EVD alone in both the fixed- and random-effect models, with RR 0.58 (95% CI 0.47–0.72) for both models (Fig. 1). Heterogeneity was low in the random-effects (RE) model (*I*^2^ = 0%, *p*-heterogeneity = 0.89). Meta-regression found no sources for confounding. Egger’s test and Begg’s test were not significant, with *p* = 0.14 and *p* = 0.15, respectively. The funnel plot showed a possible bias but correction via the trim-fill method (Supplementary Fig. 2) did not yield a significantly different model.

### Functional Outcome

**Fig. 2:**
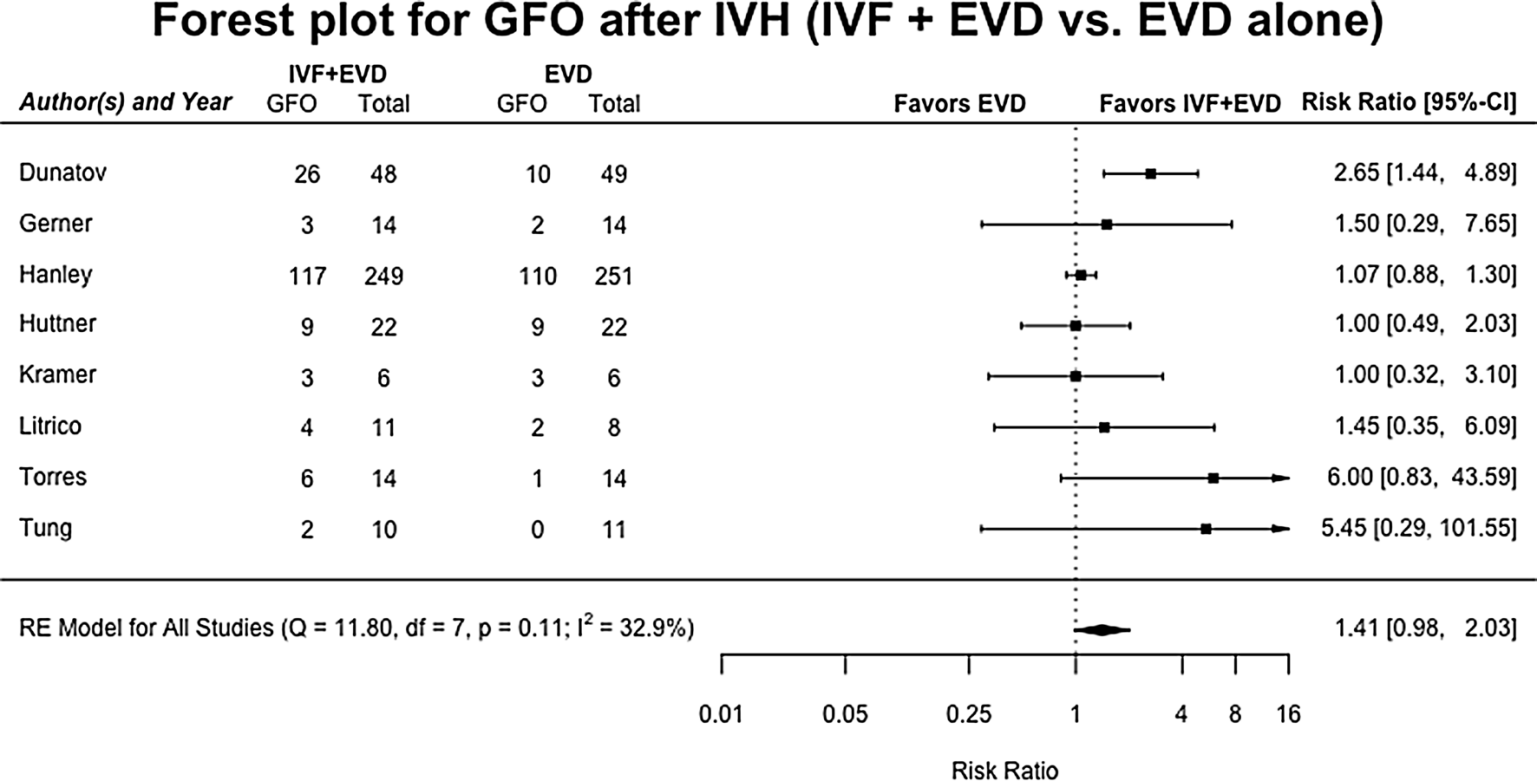
Forest plot for good functional outcome after intraventricular hemorrhage. Pooled risk ratios for good functional outcome in patients receiving IVF and EVD versus those being treated with EVD alone, in a random-effects model. It should be noted that GFO is a positive outcome, with a higher RR indicating a higher chance of this occurring in the intervention group compared to the control. Solid squares represent the point estimate of each study, with 95% CI being shown in error bars. The diamond represents the pooled estimate of the risk ratios. *I*^2^ and *p* values for heterogeneity are shown. *CI* confidence interval, *EVD* external ventricular drain, *GFO* good functional outcome, *IVF* intraventricular fibrinolysis, *IVH* intraventricular hemorrhage, *RE* random effects

Functional outcome was assessed in eight studies totaling 749 patients [12, 14, 28, 29, 32, 33, 36, 38]. GFO did not differ after IVF treatment compared to patients receiving EVD alone with RR 1.41 (95% CI 0.98–2.03), in both fixed- and random-effect models (Fig. 2). Heterogeneity of the RE model was moderate, at 32.9%, *p* = 0.11. Meta-regression analysis showed that study quality was an effect modifier with *β* = 0.83, *p* = 0.005, as was study design, with retrospective cohort studies having a *β* = 0.90, *p* = 0.006, with RCT as reference category. Curiously, the impact factor of the journal in which the study was published was also an effect modifier, with *β* = 0.02, *p* = 0.03, as was the country in which the study was done: studies in Europe modified the effect with *β* = 0.52, *p* = 0.03, compared to North-American studies (Supplementary Table 6). Egger’s test was significant at *p* = 0.04, Begg’s test was not, *p* = 0.11. The funnel plot showed possible bias in reporting, but correction via the trim-and-fill method (Supplementary Fig. 3) did not predict a significant new model, with RR 1.31 (95% CI 0.93–1.85).

### Ventriculitis

Information regarding the incidence of ventriculitis was available from 15 studies [12–14, 25–31, 33–35, 37, 38]. Ventriculitis rates were not significantly lower in patients using IVF with EVD compared to patients receiving EVD alone: 0.68 (95% CI 0.45–1.03) for both the fixed- and random-effect models, *p* = 0.06 (Supplementary Fig. 4). There was no evidence for heterogeneity in the RE model (*I*^2^ = 0%, *p* = 0.97), and no factor was identified as a source of heterogeneity through meta-regression. Egger’s test (*p* = 0.44) and Begg’s test (*p* = 0.86) were not significant. The funnel plot showed a possible indication for bias (Supplementary Fig. 5), and correction via the trim-fill method did yield a significant model, with a decreased risk of ventriculitis (RR 0.61, 95% CI 0.41–0.91, *p* = 0.02), with no heterogeneity (0%, *p* = 0.94).

### Bleeding

Symptomatic intracranial bleeding after start of therapy was evaluated in 926 patients in 14 studies [12–14, 25–28, 30, 31, 33–36, 38, 39]. The fixed- and random-effect models showed no significant impact of the treatment on outcome (RR 1.50, 95% CI 0.89–2.52), with low heterogeneity (*I*^2^: 0%, *p* = 0.99) in the RE model (Supplementary Fig. 6). Meta-regression showed that none of the factors contributed to heterogeneity. Egger’s (*p* = 0.44) and Begg’s (*p* = 0.85) tests were not significant, but the funnel plot showed possible bias (Supplementary Fig. 7). Correction for publication bias via the trim-fill method predicted a significant random-effects model with RR 1.67 (95% CI 1.01–2.74) with low heterogeneity (*I*^2^: 0%, *p* = 0.99).

### Obstruction

**Fig. 3:**
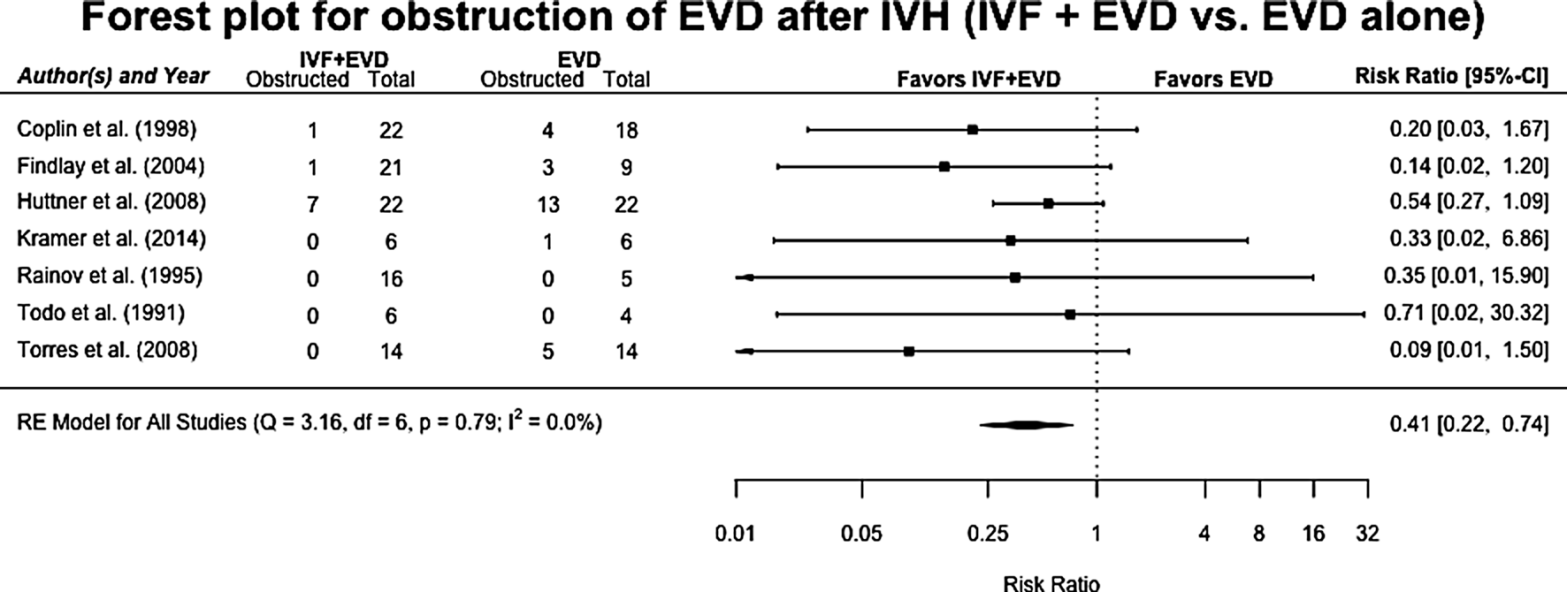
Forest plot for obstruction after intraventricular hemorrhage. Pooled risk ratios for obstruction in patients receiving IVF and EVD versus those being treated with EVD alone, in a random-effects model. Solid squares represent the point estimate of each study, with 95% CI being shown in error bars. The diamond represents the pooled estimate of the risk ratios. *I*^2^ and *p* values for heterogeneity are shown. *CI* confidence interval, *EVD* external ventricular drain, *IVF* intraventricular fibrinolysis, *IVH* intraventricular hemorrhage, *RE* random effects

EVD obstruction rate was evaluated in seven studies totaling 185 patients [26–28, 31, 33, 34, 36]. Obstruction rates were significantly lower in patients treated with IVF compared to patients receiving EVD alone with a RR of 0.41 (95% CI 0.22–0.74) in both the fixed- and random-effect models (Fig. 3). In the RE model, heterogeneity was low at *I*^2^ = 0% with *p* = 0.79. No sources of heterogeneity were identified by meta-regression. Funnel plot, Egger’s test (*p* = 0.26), and Begg’s test (*p* = 1.0) did not indicate publication bias.

### Time to IVH Resolution

**Fig. 4:**
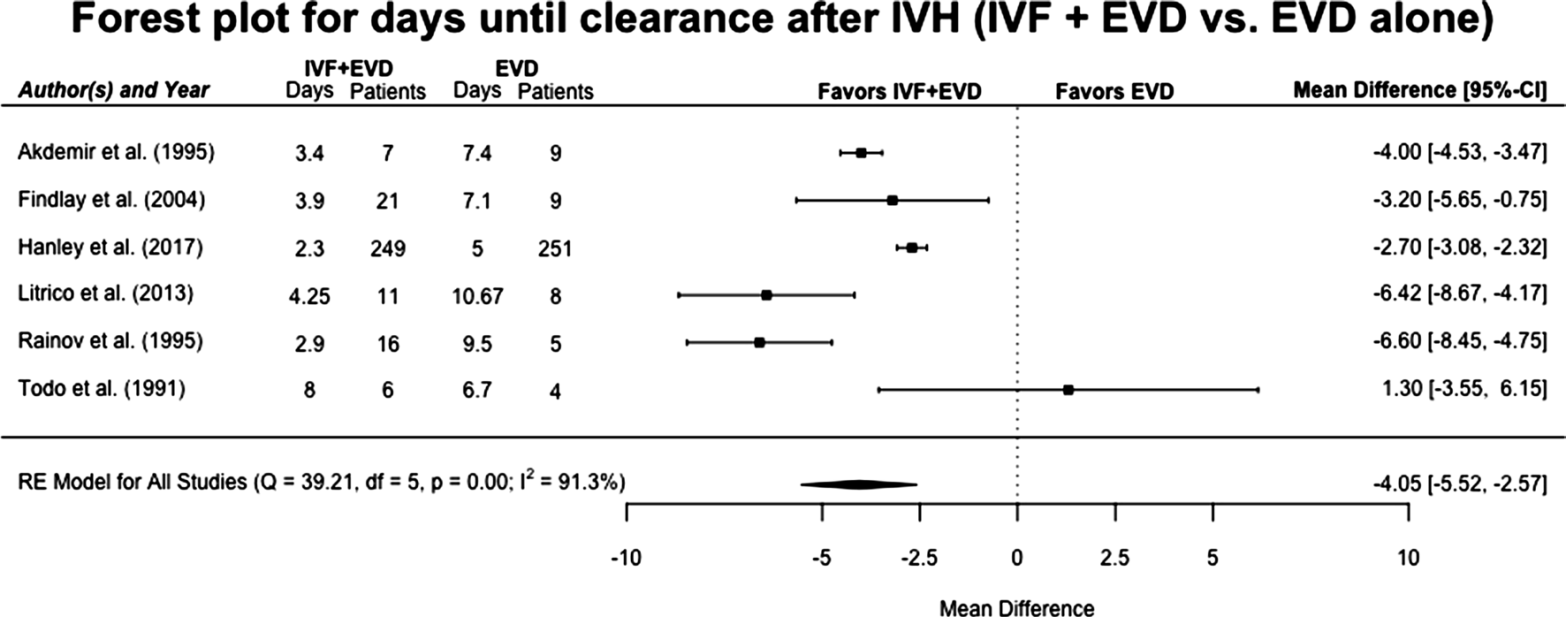
Forest plot for days until clearance of third and fourth ventricles after intraventricular hemorrhage. Mean differences for days until clearance of ventricles in patients receiving IVF and EVD versus those being treated with EVD alone, in a random-effects model. Solid squares represent the point estimate of each study, with 95% CI being shown in error bars. The diamond represents the pooled estimate of the median differences. *I*^2^ and *p* values for heterogeneity are shown. *CI* confidence interval, *EVD* external ventricular drain, *IVF* intraventricular fibrinolysis, *IVH* intraventricular hemorrhage, *RE* random effects

Clearance of the third and fourth ventricles was assessed in six studies totaling 596 patients [14, 26, 27, 30, 31, 38]. The random-effects model showed a significantly faster ventricular clearance in patients receiving IVF with EVD compared to patients receiving EVD alone (mean difference − 4.05 days, 95% CI between − 5.52 and − 2.57) (Fig. 4). The fixed-effect model showed similar, yet slightly weaker, results (mean difference − 3.27 days (95% CI between − 3.57 and − 2.97). Heterogeneity was high in the RE model, however, with *I*^2^: 91.3%, *p* < 0.0001. Study country (Europe: *β* = −3.82, *p* < 0.001, Middle East: *β* = −1.29, *p* = 0.0001, North America as reference), design (case–control: *β* = −2.81, *p* = 0.035, RCT as reference), size (*β* = 0.003, *p* < 0.0001), and the impact factor of the journal in which the study was published (*β* = 0.03, *p* < 0.0001) all interfered with outcome, as did patient age (*β* = 0.46, *p* < 0.0001) (Supplementary Table 6). The funnel plot, Egger’s test (0.52), and Begg’s test (0.72) indicated low possibility of bias.

### Shunt Dependency

Shunt dependency after IVH was assessed in 16 studies [12–14, 26, 27, 29–36, 38, 39]. There was no difference between IVF + EVD and EVD alone in the risk shunt dependency using the random-effect or fixed-effect models (RR 0.93, 95% CI 0.70–1.22, *p* = 0.59). No heterogeneity was found in the RE model, *I*^2^: 0%, *p* = 0.71. Average age was identified as a possible source of heterogeneity, with *β* = 0.11 (*p* = 0.048) (Supplementary Table 6). Egger’s and Begg’s tests were not significant, with *p* = 0.27 and *p* = 0.33, respectively. The funnel plot showed possible publication bias (Supplementary Fig. 8), but correction via the trim-fill method did not yield a significantly different model.

### IVF in SAH Versus IPH

Since SAH and IPH have very different underlying pathologies, we analyzed differences between these patient groups. The origin of the IVH (IPH or SAH) showed no significant impact on any of the outcomes in our meta-regression analysis (Supplementary Table 6). We performed a subgroup analysis to further evaluate this finding, comparing IPH only studies (12 studies, 885 patients) with SAH only studies (five studies, 109 patients). Risk of mortality, obstruction, and clearance of the ventricles remained significantly improved in IPH patients receiving IVF, while only the risk of faster clearance of the ventricles was significantly improved in SAH patients with IVF (data not shown).

## Discussion

Our findings showed that patients receiving IVF for non-traumatic IVH had a lower risk of mortality and EVD obstruction, and a faster clearance of blood from the third and fourth ventricles compared to patients receiving only an EVD. There was no significant difference in GFO, shunt dependency, ventriculitis, and bleeding. However, in a sensitivity analysis, correction for publication bias estimated an increased risk of intracranial bleeding and a lower risk of ventriculitis for patients receiving IVF.

Previous systematic reviews have been cautiously optimistic regarding the use of IVF in IVH [16, 17, 40]. Recently, a systematic review from Wang et al. [16] concluded that IVF was associated with lower risk of mortality (RR 0.63, 95% CI 0.47–0.83) and ventriculitis (RR 0.57, 95% CI 0.35–0.93), but did not change the likelihood of poor functional outcome (RR 0.96, 95% CI 0.83–1.11), shunt dependence (RR 1.06, 95% CI 0.75–1.49), or re-hemorrhage (RR 0.57, 95% CI 0.35–0.93). At the same time, Baker et al. [17] published a systematic review regarding IVF in IPH specifically, and they concluded that IVF-treated patients had an increased likelihood of GFO (RR 0.67, 95% CI 0.55–0.83), with ‘GFO’ defined as having a GOS of 3 or higher. We felt that a GFO should be defined as GOS 4 or higher, as GOS 3 is defined as ‘severe injury with permanent need for help with daily living’ [41]. Our analysis showed no difference in GFO, albeit there was a trend toward improved GFO after IVF. Publication bias was not assessed for GFO by Baker et al. or for any outcome by Wang et al., while our funnel plot with trim-fill analysis indicated the presence of this. However, correction via the trim-fill method did not predict a different model, but did lessen the trend observed in the uncorrected model. We included more patients than previous systematic reviews. Due to our strict inclusion criteria for the purpose of reducing heterogeneity, some studies previously reviewed in other meta-analyses were excluded from our analysis (see Supplementary Table 7). The findings from our meta-analysis aligned with the results of the CLEAR-III trial [14]: Similar outcomes were observed with respect to mortality (hazard ratio 0.60, 95% CI 0.41–0.86) and GFO (RR 1.06, 95% CI 0.88–1.28). Risk of ventriculitis (RR 0.55, 95% CI 0.64–0.90) was lower in their study, which underscores our results from the trim-fill analysis.

An interesting find was the predicted increase in symptomatic hemorrhages in patients receiving IVF, after trim-fill correction for publication bias. The number of these events was small in most studies, with two studies reporting much higher incidences of hemorrhage post-treatment than the average [25, 35]. Both examined IVF specifically in IPH and used rt-PA at a dose of 6 mg/24 h. This is above the median of 4 mg/24 h for all (r)t-PA studies, although Ducruet et al. [35] lowered their dosing during the study. The trim-fill analysis indicated that this serious complication might be underestimated because of publication bias. However, recent studies, such as the CLEAR-III trial, have been developed with stringent safety criteria in place to prevent complications, such as clot stability confirmed on CT scan at least 6 h after EVD placement, low doses spread over 24 h, and daily CT scans to detect complications early [14]. These studies provide better precautions to prevent hemorrhage compared to earlier studies which has increased the safety of IVF use. Future work should stress the importance of these precautions and provide further data on the risk of symptomatic bleeding after IVF.

Despite the obvious differences in underlying pathology, only minor differences in outcomes were observed for IVF in IVH between SAH and IPH. There were fewer studies evaluating the use of IVF in SAH, and thus, not all results were significant, but similar trends were observed. It is important to note that this does not mean outcomes are similar between IPH and SAH, but that IVF does not seem to have a different effect in these different pathologies. Given the small number of studies investigating IVF in SAH, it could be that more nuanced differences were missed in this meta-analysis. Currently, the FIVHeMA study (Intraventricular Fibrinolysis for Aneurysmal SAH, clinicaltrials.gov: NCT03187405) is recruiting patients, which will provide further insights into the use of fibrinolytics for IVH in SAH.

To our knowledge, this is the first systematic review and meta-analysis on this subject that employs meta-regression and assesses publication bias via the trim-fill method. The meta-regression analysis allowed us to analyze the influence of multiple variables on the outcomes after IVH. Not only did we account for different study variables such as quality, region of study, study size, and impact factor of the journal, we were also able to assess the impact of the origin of the IVH and the type of treatment received, although it was not possible to account for dosage. The type of fibrinolytic used did not appear to impact outcomes, contrary to previous publications [16, 40].

Finally, this is the first meta-analysis in which obstruction and time until clearance of ventricles, both important variables for clinicians working with EVD, have been evaluated in relation to IVF.

Previous studies observed a decrease in ventriculitis with the use of IVF [14, 16, 40]. In our meta-analysis, we observed a clear trend, but the difference was not significant. However, possible publication bias was observed and correction via the trim-fill method predicted a significant model with a RR that was in line with results from the CLEAR-III trial, which showed an RR of 0.55, 95% CI 0.31–0.97 [14]. Interestingly, the risk of ventriculitis was shown to decrease, even though repeatedly accessing the ventricles is commonly regarded as increasing the risk of ventriculitis and is commonly avoided in the clinic [18, 42]. This protective association against ventriculitis could probably be attributed to the faster clearance of the ventricles, limiting the time an EVD is in situ, which is also directly related to increased incidence of ventriculitis [4]. Apparently, this outweighs the risk of accessing the EVD repeatedly and injecting medication. The CLEAR-III trial [14] used a saline control, while in other trials the EVDs of control patients were not accessed [26, 27, 30, 38, 43], further highlighting the positive effects of IVF therapy on ventriculitis rates.

Staykov et al. recently showed the benefits of combining IVF with lumbar drainage in patients with IPH-related IVH regarding shunt dependency [43]. None of the 14 included patients treated with IVF and lumbar drainage developed the need for permanent shunting compared to 7 of 16 (44%) patients treated with IVF alone, *p* = 0.007. Lumbar drainage was outside the scope of this review, but as shunt dependency is an important outcome for patients and clinicians, we evaluated this in our analysis. We did not observe differences in shunt dependency between the two groups. It must be noted, however, that the criteria and thresholds to place permanent shunting varied between studies and were often poorly described, limiting generalizability and pooling for this outcome. Not only did indications for this procedure differ among institutions, they also varied within hospitals and studies, as the timing of permanent shunt placement was generally left to the treating physician. We suggest that future studies provide unified protocols regarding the timing and indication of permanent shunt surgery, and describe this in their papers. This will increase comparability among studies and generalizability of results. The use of lumbar drainage and IVF as described by Staykov et al. also warrants further investigation.

Our study had several limitations. Via the trim-fill method, we estimated the number of ‘missing studies’ due to publication bias in our meta-analysis and were able to provide a prediction of the effect that these studies would have had on the overall outcome [44]. This showed an increased risk of intracranial hemorrhage and a decreased risk of ventriculitis. Nevertheless, it must be noted that the trim-fill method is a statistical model which provides an *estimate* of the effect of publication bias and cannot provide the true effect it has had on the results. It has been shown to reduce bias in pooled estimates, especially when between-study heterogeneity exists [45], yet it remains a statistical model based on assumptions. Although both of our outcomes had low between-study heterogeneity, results derived from this method should still be considered as estimates and do not replace actual patient data.

The studies included in this review consisted not only of RCTs but also of retrospective and case–control studies. The study quality varied widely between included studies and was moderate on average. Studies like the CLEAR-III trial or by Gerner et al., well designed and performed RCTs, were of much higher quality than most previous trials, and combining their results was not ideal. Most RCTs had a high or unclear risk of bias. Due to the limited number of RCTs available, we opted to include observational studies and employ meta-regression to account for these differences in study quality, size, and design.

Functional outcome was determined at different time points in different studies, with some noting it at discharge, while others after 3 or 6 months. For GFO, we included studies describing functional outcome 3 months or more after initial admission, with time of assessment ranging between 3 and 12 months. Ideally, all studies would assess this outcome at similar time points, for it is possible that longer rehabilitation might benefit one intervention over the other.

To fully evaluate the place of IVF in treating IVH, more data regarding other outcomes are needed; length of intensive care unit stay, length of hospital stay, time until permanent shunt placement, Graeb scores, and Glasgow Coma Scale score on admission are all relevant measures in evaluating the effect of IVF, but have so far been recorded infrequently and in varying details.

In this systematic review and meta-analysis, patients treated with IVF after IVH showed a decreased risk for mortality, fewer obstructions of the external ventricular drains and faster clearance of the ventricles. Functional outcome after 3 months did not differ, as did the risk for shunt dependency. After correcting for publication bias, a possible increase in the risk of symptomatic intracranial hemorrhage and a decrease in the risk of ventriculitis was suggested in patients receiving IVF. In our opinion, the benefits of IVF are not necessarily in improving outcomes, but more in aiding external drain management and possibly preventing ventriculitis. However, more data is needed to fully elucidate these effects and to determine the exact place for IVF in the treatment of IVH.

## Electronic supplementary material

Below is the link to the electronic supplementary material.

Supplementary material 1 (DOCX 9530 kb)
