# Supplementary material for: Fibrinolytics and Intraventricular Hemorrhage: A Systematic Review and Meta-analysis
Source: Neurocrit Care. 2019 Aug 2;32(1):262–71. doi: 10.1007/s12028-019-00786-5 (PMC7012971; doi:10.1007/s12028-019-00786-5)
Supplement: Supplementary file 1 — Supplementary material 1 (DOCX 9530 kb) [file 12028_2019_786_MOESM1_ESM.docx]

**Supplementary**

**Fibrinolytics and Intraventricular Hemorrhage: A systematic review and meta-analysis**

Thomas S. van Solinge MD^1,2,3^, Ivo S. Muskens MD^1^, Vasileios Kavouridis MD^1^, William B. Gormley MD PhD MBA^1^, Rania A. Mekary PhD ,MSc, MSc^1,4^, Marike L.D. Broekman MD PhD JD^1,2,3,5^, Omar Arnaout MD^1^.

^1^Computational Neurosciences Outcome Center, Brigham and Women’s Hospital, Harvard Medical School, Boston, MA, USA

^2^Department of Neurology, Massachusetts General Hospital, Harvard Medical School, Boston, MA, USA.

^3^Department of Neurosurgery, Leiden University Medical Center, Leiden, The Netherlands

^4^Department of Pharmaceutical Business and Administrative Sciences, School of Pharmacy, MCPHS University, Boston, MA, USA

^5^Department of Neurosurgery, Haaglanden Medical Center, The Hague, The Netherlands.

Correspondence:

Thomas S. van Solinge, MD

PhD-student

Department of Neurosurgery, LUMC

Albinusdreef 2, Leiden, 2333 ZA

The Netherlands

tsolinge@planet.nl

**Supplementary Methods**

*Statistical analysis*

Using meta-regression analysis, the following study characteristics were analyzed for possible heterogeneity: age (mean), study design (RCT, cohort study, case-control), region (Asia, Europe, N-America, Middle East), origin of IVH (SAH, IPH, or IPH & SAH), treatment (urokinase or t-PA), journal impact factor, study size, and study quality (high or low). If no mean value was specified, but median and range values were, the mean was calculated using the methods specified by Hozo et al^1^. If median and inter quartile range were available, mean and standard deviation were estimated using the methods described by Wan et al^2^.

Funnel plots, Egger’s linear regression test and Begg’s correlation test were used for evaluation of publication bias^3,4^ and adjustment was done by the trim and fill method when publication bias was present^5^.

Other forms of bias considering RCTs were assessed via the Cochrane method.

**Supplementary Results**

Study details

Ten studies indicated the Glasgow Coma Score (GSC) on admission and nine studies indicated the Graeb-scores (Table 2). Mean GCS at arrival was 7.5 (median: 7.2), with similar means recorded for IVF (7.9, median 7.1) and EVD only patients (7.8, median 7.3). Mean Graeb score for IVF patients was 9.2 (median: 8.3), and 8.3 (median 9.1) for EVD patients upon first assessment. Additional details regarding patient data and outcomes can be found in Supplementary Tables 3 and 4.

The median score for the quality of the retrospective studies was 6 points on the NOS-scale, resulting in 7 out of 12 studies being labeled as ‘high-quality’. In the RCTs, the median score on the JADAD-scale was 3.5, with 4 out of 8 studies being considered of high quality, Table 1. Risk for bias was high in RCTs by Akdemir et al, Kramer et al, Litrico et al, Naff et al (2004) and Tung et al 1998, low in the study by King et al, and unclear in Hanley et al and Naff (2011), Supplementary Table 5.

**Supplementary references**

1. Hozo SP, Djulbegovic B, Hozo I. Estimating the mean and variance from the median, range, and the size of a sample. *BMC Med Res Methodol*. 2005;5(1):13. doi:10.1186/1471-2288-5-13.

2. Wan X, Wang W, Liu J, Tong T. Estimating the sample mean and standard deviation from the sample size, median, range and/or interquartile range. *BMC Med Res Methodol*. 2014;14:135. doi:10.1186/1471-2288-14-135.

3. Begg CB, Mazumdar M. Operating characteristics of a rank correlation test for publication bias. *Biometrics*. 1994;50(4):1088-1101.

4. Egger M, Davey Smith G, Schneider M, Minder C. Bias in meta-analysis detected by a simple, graphical test. *BMJ*. 1997;315(7109):629-634.

5. Duval S, Tweedie R. Trim and fill: A simple funnel-plot-based method of testing and adjusting for publication bias in meta-analysis. *Biometrics*. 2000;56(2):455-463.

6. Etminan N, Beseoglu K, Eicker SO, Turowski B, Steiger HJ, H??nggi D. Prospective, randomized, open-label phase ii trial on concomitant intraventricular fibrinolysis and low-frequency rotation after severe subarachnoid hemorrhage. *Stroke*. 2013;44(8):2162-2168. doi:10.1161/STROKEAHA.113.001790.

7. Fabiano AJ, Gruber TJ, Baxter MS, A.J. F, T.J. G, M.S. B. Increased ventriculostomy infection rate with use of intraventricular tissue plasminogen activator: A single-center observation. *Clin Neurol Neurosurg*. 2013;115(11):2362-2364. doi:10.1016/j.clineuro.2013.08.018.

8. Gubucz I, Kakuk I, Major O, et al. [Effectiveness and safety of intraventricular fibrinolysis in secondary intraventricular hemorrhages (a prospective, randomized study)]. *Orv Hetil*. 2004;145(31):1609-1615.

9. Naff NJ, Carhuapoma JR, Williams MA, et al. Treatment of intraventricular hemorrhage with urokinase : effects on 30-Day survival. *Stroke*. 2000;31(4):841-847.

10. Ramakrishna R, Sekhar LN, Ramanathan D, et al. Intraventricular tissue plasminogen activator for the prevention of vasospasm and hydrocephalus after aneurysmal subarachnoid hemorrhage. *Neurosurgery*. 2010;67(1):110-117. doi:10.1227/01.NEU.0000370920.44359.91.

11. Tush GM, Tesoro EP, Prabhu S, Brown K BM. ntraventricular urokinase for the treatment of intraventricular hemorrhage (abstract). *Crit Care Med*. 1999;27(1):79.

12. Volbers B, Wagner I, Willfarth W, Doerfler A, Schwab S, Staykov D. Intraventricular fibrinolysis does not increase perihemorrhagic edema after intracerebral hemorrhage. *Stroke*. 2013;44(2):362-366. doi:10.1161/STROKEAHA.112.673228.

**Supplementary tables**

| Supplementary Table 1: search strategy |
| --- |
| Medline |
| (((((intraventric*[Title/Abstract] OR ventric*[Title/Abstract]) AND (Cerebr*[Title/Abstract] OR intracerebral[Title/Abstract] OR brain[Title/Abstract] OR intracranial[Title/Abstract] OR "Brain"[Mesh:NoExp] OR "Cerebrum"[Mesh])) OR “Cerebral Ventricles”[Mesh]))) AND ((hemorrhag*[Title/Abstract] OR haemorrhag*[Title/Abstract] OR bleed*[Title/Abstract] OR blood[Title/Abstract] OR haematom*[Title/Abstract] OR hematoma*[Title/Abstract] OR clot*[Title/Abstract] OR thrombus[Title/Abstract] OR thrombi[Title/Abstract] OR "Hemorrhage"[Mesh] OR "Hematoma"[Mesh] OR "Cerebral Hemorrhage"[Mesh])) AND (Thrombol*[Title/Abstract] OR blood clot lysis[Title/Abstract] OR infusion[Title/Abstract] OR injection[Title/Abstract] OR alteplase[Title/Abstract] OR urokinase[Title/Abstract] OR tenecteplase[Title/Abstract] OR Streptokinase [Title/Abstract] OR Plasminogen Activator*[Title/Abstract] OR "Thrombolytic Therapy"[Mesh] OR "Tissue Plasminogen Activator"[Mesh] OR "Infusions, Intraventricular"[Mesh] OR "Injections, Intraventricular"[Mesh] OR "Fibrinolytic Agents"[Mesh] OR "Fibrinolytic Agents" [Pharmacological Action] OR "Plasminogen Activators"[Mesh]) |
|  |
| Embase |
| ('brain ventricle'/exp OR ((intraventric*:ab,ti OR ventric*:ab,ti) AND (brain:ab,ti OR 'brain'/de OR 'forebrain'/exp OR 'intracranial':ab,ti OR cerebr*:ab,ti))) AND (bleed*:ab,ti OR hemorrhag*:ab,ti OR haemorrhag*:ab,ti OR hematom*:ab,ti OR haematom*:ab,ti OR clot*:ab,ti OR thrombus:ab,ti OR thrombi:ab,ti OR 'brain hemorrhage'/exp OR 'thrombus'/de OR 'bleeding'/de OR 'hematoma'/de OR 'brain hematoma'/exp OR 'traumatic hematoma'/de) AND ('blood clot lysis':ab,ti OR thrombol*:ab,ti OR 'infusion':ab,ti OR 'injection':ab,ti OR 'alteplase':ab,ti OR 'urokinase':ab,ti OR 'tenecteplase':ab,ti OR plasminogen activator*:ab,ti OR 'intracerebroventricular drug administration'/de OR 'fibrinolytic therapy'/de OR 'fibrinolysis'/exp OR 'plasminogen activator'/exp) |

| Supplementary Table 2: Patients’ Characteristics on Admission | | | | | | | |
| --- | --- | --- | --- | --- | --- | --- | --- |
| Study | Male, *n (%)* | Age, *mean (SD)* | | Graeb score, *mean (SD)* | | GCS, *mean (SD)* | |
|  |  | IVF+EVD | EVD | IVF+EVD | EVD | IVF+EVD | EVD |
| Akdemir, 1995 | 5 (31.3) | 61 (5) | 53 (12) | 9.8 (0.4) | 10.3 (0.4) | 7.1 (1.1) | 6.8 (1.3) |
| Coplin, 1998 | 17 (42.5) | 57 (15) | 56 (15) | *NA* | NA | 7 (2.9) | 6.2 (2.5) |
| Ducruet, 2010 | 26 (86.7) | 66 (4) | 56 (4) | 8.7 (1.8) | 8.1 (2.1) | 5.7 (3.3) | 5.0 (5.7) |
| Dunatov, 2011 | 58 (59.8) | 58 | 59 | *NA* | *NA* | 12 | 11.5 |
| Findlay, 2004 | 25 (83.3) | 55 (13) | 57 (13) | 9.6 (1.8) | 9.1 (2) | 9.9 (2.6) | 11.3 (1.5) |
| Gerner, 2014 | 24 (85.7) | 54 (11) | 53 (13) | 7 (1.6) | 2.7 (2.3) | *NA* | *NA* |
| Hallevi, 2011 | *NA* | 55 (11) | 61 (16) | *NA* | *NA* | *NA* | *NA* |
| Hanley, 2017 | 278 (55.6) | 59 (4) | 59 (4) | *NA* | *NA* | *NA* | *NA* |
| Huttner, 2008 | 27 (61.4) | *NA* | *NA* | 8.3 (3.1) | 8 (2.3) | 7 (2.6) | 7.8 (2.9) |
| King, 2012 | 10 (62.5) | 58 | 54 | *NA* | *NA* | *NA* | *NA* |
| Kramer, 2014 | 3 (25.0) | 56 (3) | 63 (8) | *NA* | *NA* | *NA* | *NA* |
| Litrico, 2013 | 12 (63.2) | 52 (11) | 60 (7) | 10.3 (1.1) | 10.9 (0.6) | 8 (2.8) | 7.3 (3.5) |
| Naff, 2004 | 8 (72.7) | 50 (15) | 55 (5) | *NA* | *NA* | 7.1 (3.8) | 7.8 (3.7) |
| Naff, 2011 | 26 (54.2) | 54 (2) | 57 (2) | *NA* | *NA* | *NA* | *NA* |
| Rainov, 1995 | 10 (47.6) | 51 (10) | 53 (5) | 8.5 (1.5) | 9 (1.6) | *NA* | *NA* |
| Todo, 1991 | 8 (80.0) | 52 (17) | 50 (26) | 10 (1.5) | 9.8 (0.8) | 6.2 (1.9) | 5.2 (1.6) |
| Torres, 2008 | 13 (46.4) | 57 | 55 | 11 | 10 | *NA* | *NA* |
| Tung, 1998 | 8 (38.1) | 62 (7) | 57 (15) | *NA* | *NA* | *NA* | *NA* |
| Varelas, 2005 | 12 (60.0) | 51 (11) | 51 (7) | 8.5 (2.3) | 5.3 (3) | 8.6 (3.7) | 9.2 (4.0) |
| EVD: extra ventricular drain. IVF: intra ventricular fibrinolysis. GCS: Glasgow Coma Scale. NA: not available. SD: standard deviation. | | | | | | | |

| Supplementary Table 3: Patients’ Outcomes | | | | | | | | | | |
| --- | --- | --- | --- | --- | --- | --- | --- | --- | --- | --- |
| Study | Mortality, *n (%)* | | Ventriculitis, *n (%)* | | Bleeding, *n (%)* | | Obstruction, n (%) | | Time until clearance, days, *mean (SD)* | |
|  | IVF+EVD | EVD | IVF+EVD | EVD | IVF+EVD | EVD | IVF+EVD | EVD | IVF+EVD | EVD |
| Akdemir, 1995 | 2 (28.6) | 6 (66.7) | 0 (0.0) | 1 (11.1) | 0 (0.0) | 0 (0.0) | *NA* | *NA* | 3.4 (0.5) | 7.4 (0.6) |
| Coplin, 1998 | 7 (31.8) | 12 (66.7) | 4 (18.2) | 4 (22.2) | 3 (13.6) | 1 (5.6) | 1 (4.5) | 4 (22.2) | *NA* | *NA* |
| Ducruet, 2010 | 4 (30.8) | 7 (41.2) | 1 (7.7) | 1 (5.9) | 5 (38.5) | 5 (29.4) | *NA* | *NA* | *NA* | *NA* |
| Dunatov, 2011 | 5 (10.4) | 15 (30.6) | 2 (4.2) | 0 (0.0) | 1 (2.1) | 0 (0.0) | *NA* | *NA* | *NA* | *NA* |
| Findlay, 2004 | 1 (4.8) | 1 (11.1) | 0 (0.0) | 1 (11.1) | 3 (14.3) | 1 (11.1) | 1 (4.8) | 3 (33.3) | 3.9 (1.0) | 7.1 (3.7) |
| Gerner, 2014 | *NA* | *NA* | *NA* | *NA* | *NA* | *NA* | *NA* | *NA* | *NA* | *NA* |
| Hallevi, 2011 | 3 (16.7) | 1 (9.1) | *NA* | *NA* | *NA* | *NA* | *NA* | *NA* | *NA* | *NA* |
| Hanley, 2017 | 46 (18.5) | 73 (29.1) | 17 (6.8) | 31 (12.4) | 6 (2.4) | 5 (2.0) | *NA* | *NA* | 2.3 (0.8) | 5 (3.0) |
| Huttner, 2008 | 7 (31.8) | 8 (36.4) | *NA* | *NA* | 0 (0.0) | 0 (0.0) | 7 (31.8) | 13 (59.1) | *NA* | *NA* |
| King, 2012 | 1 (14.3) | 4 (44.4) | 1 (14.3) | 1 (11.1) | *NA* | *NA* | *NA* | *NA* | *NA* | *NA* |
| Kramer, 2014 | 1 (16.7) | 2 (33.3) | 1 (16.7) | 1 (16.7) | 0 (0.0) | 1 (16.7) | 0 (0.0) | 1 (16.7) | *NA* | *NA* |
| Litrico, 2013 | 5 (45.5) | 5 (62.5) | 2 (18.2) | 2 (25.0) | 1 (9.1) | 0 (0.0) | *NA* | *NA* | 4.25 (1.8) | 10.7 (2.9) |
| Naff, 2004 | 0 (0.0) | 1 (20.0) | *NA* | *NA* | 0 (0.0) | 0 (0.0) | *NA* | *NA* | *NA* | *NA* |
| Naff, 2011 | 5 (19.2) | 5 (22.7) | 2 (7.7) | 2 (9.1) | 11 (42.3) | 3 (13.6) | *NA* | *NA* | *NA* | *NA* |
| Rainov, 1995 | 0 (0.0) | 1 (20.0) | 0 (0.0) | 0 (0.0) | 0 (0.0) | 0 (0.0) | 0 (0.0) | 0 (0.0) | 2.9 (0.7) | 9.5 (2.1) |
| Todo, 1991 | 0 (0.0) | 2 (50.0) | 0 (0.0) | 0 (0.0) | 0 (0.0) | 0 (0.0) | 0 (0.0) | 0 (0.0) | 8.0 (3.3) | 6.7 (4.2) |
| Torres, 2008 | 4 (28.6) | 8 (57.1) | 1 (7.1) | 2 (14.3) | 0 (0.0) | 0 (0.0) | 0 (0.0) | 5 (35.7) | *NA* | *NA* |
| Tung, 1998 | 1 (10.0) | 7 (63.6) | 2 (20.0) | 1 (9.1) | *NA* | *NA* | *NA* | *NA* | *NA* | *NA* |
| Varelas, 2005 | 1 (10.0) | 4 (40.0) | 0 (0.0) | 0 (0.0) | 0 (0.0) | 0 (0.0) | *NA* | *NA* | *NA* | *NA* |
| EVD: extra ventricular drain. IVF: intra ventricular fibrinolysis. NA: not available. SD: standard deviation. | | | | | | | | | | |

| Supplementary Table 4: Patients’ Outcomes | | | | | | |
| --- | --- | --- | --- | --- | --- | --- |
| Study | Good functional outcome, *n(%) [time of assessment]* | | Shunt dependency, n (%) | | Length of stay, days  *mean (SD) [location]* | |
|  | IVF+EVD | EVD | EVF+EVD | EVD | IVF+EVD | EVD |
| Akdemir, 1995 | *NA* | *NA* | 1 (14.2) | 3 (33.3) | *NA* | *NA* |
| Coplin, 1998 | 8 (36.4)[discharge] | 4 (22.2) | 4 (18.1) | 2 (11.1) | 40.8 (29.0) [hospital] | 37.5 (34.7) |
| Ducruet, 2010 | *NA* | *NA* | 5 (38.5) | 1 (5.9) | 21.3 (14.1) [hospital] | 19.4 (15.7) |
| Dunatov, 2011 | 26 (54.2) [3 months] | 10 (20.4) | 1 (2.1) | 2 (4.1) | 42.4 [hospital] | 58 |
| Findlay, 2004 | *NA* | *NA* | 4 (19.0) | 3 (33.3) | *NA* | *NA* |
| Gerner, 2014 | 3 (21.4) [3 months] | 2 (14.3) | 4 (28.6) | 4 (28.6) | 31.3 (28.7) [hospital] | 22.7 (15.8) |
| Hallevi, 2011 | *NA* | *NA* | *NA* | *NA* | *NA* | *NA* |
| Hanley, 2017 | 117 (47.0) [6 months] | 110 (43.8) | 46 (18.5) | 44 (17.5) | 14 [ICU] | 15 |
| Huttner, 2008 | 9 (40.9) [12 months] | 9 (40.9) | 4 (18.2) | 6 (27.3) | 16.2 (7.4) [ICU] | 18.1 (6.9) |
| King, 2012 | *NA* | *NA* | 1 (14.3) | 2 (22.2) | 10.1 [ICU] | 7.3 |
| Kramer, 2014 | 3 (50.0) [6 months] | 3 (50.0) | 2 (33.3) | 2 (33.3) | *NA* | *NA* |
| Litrico, 2013 | 4 (36.4) [12 months] | 2 (25.0) | 4 (36.4) | 2 (25.0) | *NA* | *NA* |
| Naff, 2004 | *NA* | *NA* | 0 | 1 (20.0) | *NA* | *NA* |
| Naff, 2011 | *NA* | *NA* | *NA* | *NA* | *NA* | *NA* |
| Rainov, 1995 | *NA* | *NA* | 1 (6.3) | 2 (40.0) | *NA* | *NA* |
| Todo, 1991 | *NA* | *NA* | 2 (33.3) | 2 (50.0) | *NA* | *NA* |
| Torres, 2008 | 6 (42.9) [6 months] | 1 (7.1) | *NA* | *NA* | *NA* | *NA* |
| Tung, 1998 | 2 (20.0) [3 months] | 0 (0.0) | 3 (30.0) | 3 (27.3) | *NA* | *NA* |
| Varelas, 2005 | 3 (30.0) [NA] | 3 (30.0) | 2 (20.0) | 5 (50.0) | 20.2 (9.2) [hospital] | 23.5 (18.69) |
| EVD: extra ventricular drain. ICU: intensive care unit. IVF: intra ventricular fibrinolysis. NA: not available. SD: standard deviation. | | | | | | |

**Table 5: Assessment of Bias in RCT studies**

Legend:


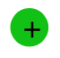
:Low risk of bias


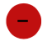
: High risk of bias


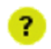
: Unclear

| Supplementary Table 6: results meta-regression analysis | | | | | | | | |
| --- | --- | --- | --- | --- | --- | --- | --- | --- |
| Outcome | Country^1^ | Design^2^ | Origin^3^ | Age | IF | Size | Treatment^4^ | Quality5 |
| Mortality | *NS* | *NS* | NS | *NS* | *NS* | *NS* | *NS* | *NS* |
| Ventriculitis | *NS* | *NS* | *NS* | *NS* | *NS* | *NS* | *NS* | *NS* |
| Bleeding | *NS* | *NS* | *NS* | *NS* | *NS* | *NS* | *NS* | *NS* |
| Obstruction | *NS* | *NS* | *NS* | *NS* | *NS* | *NS* | *NS* | *NS* |
| Time until IVH resolution | Europe: β=-3.82, p<0.001,  Middle-east: β=-1.29 p=0.0001 | Case-control: β=-2.81, p=0.035 | *NS* | β=0.46, p<0.0001 | β=0.03, p<0.0001 | β=0.003, p<0.0001 | *NS* | *NS* |
| Shunt dependency | *NS* | *NS* | *NS* | β=0.11, p=0.048 | *NS* | *NS* | *NS* | *NS* |
| Functional Outcome | *Europe:* β=0.52, p=0.03 | Retrospective cohort study:  β=0.90, p=0.006 | *NS* | *NS* | β=0.02, p=0.03 | *NS* | *NS* | β=0.83, p=0.005 |
| NS: not significant. ^1^North-America as reference,  ^2^Randomized Controlled Trial as reference, ^3^Intra Parenchymal Hemorrhage as reference,  ^4^Urokinase as reference,  ^5^High Quality as reference | | | | | | | | |

| Supplementary Table 7: excluded studies from previous meta-analysis | |
| --- | --- |
| Study | Reason for exclusion |
|  |  |
| Etminan^6^, 2013 | Combination of IVF and rotational therapy as intervention. |
| Fabiano^7^, 2013 | Heterogeneity of origin of IVH (trauma and tumor associated hemorrhages also included) |
| Gubucz^8^, 2004 | Article published in Hungarian |
| Naff^9^, 2000 | Unclear allocation of patients to control/intervention |
| Ramakrishna^10^, 2010 | Combination of IVF and lumbar drainage as intervention |
| Tush^11^, 1999 | Availability of a poster abstract only |
| Volbers^12^, 2013 | Only half of control patients receiving EVD treatment |
| EVD: extra ventricular drain. IVH: intraventricular hemorrhage | |

**Supplementary Figures**

Supplementary Figure 1

Supplementary Figure 1: PRISMA Flow Diagram of Included Studies

**Supplementary Figure 2**

**
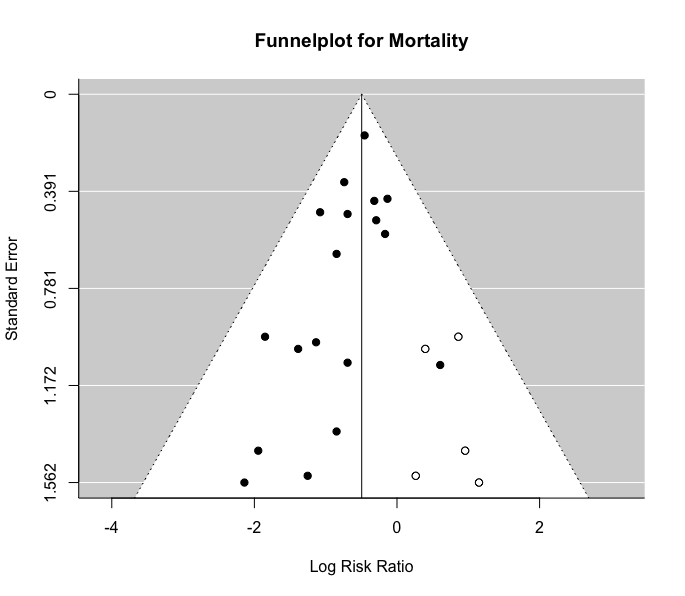
**

**Supplementary Figure 2:** Funnel plot for mortality after Trim and Fill.

• included studies. ° ‘missed’ studies indicated by Trim and Fill method.

**Supplementary Figure 3**

**
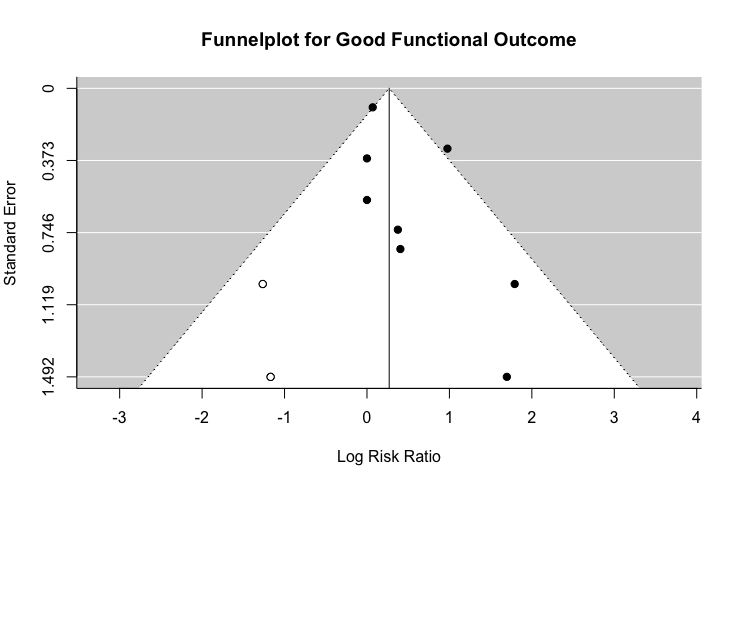
**

**Supplementary Figure 3:** Funnel plot for good functional outcome, after Trim and Fill.

• included studies. ° ‘missed’ studies indicated by Trim and Fill method.

**Supplementary Figure 4**


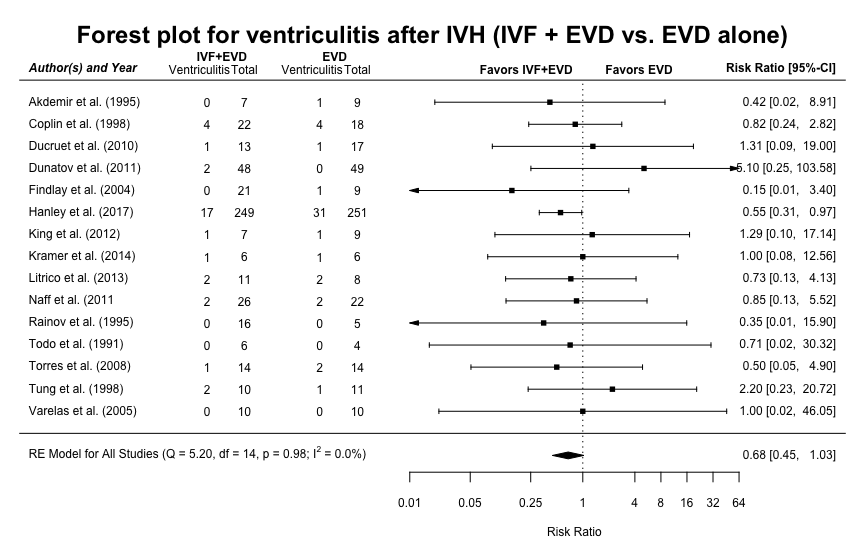


**Supplementary Figure 4:** Forest Plot for Ventriculitis after Intraventricular Hemorrhage.

Forest plot shows the pooled risk ratio for ventriculitis after IVH for patients receiving IVF and EVD, and those being treated with EVD alone using a random-effects model. Solid squares represent the point estimate of each study, with 95%-CI being shown in error bars. The diamond represents the pooled estimate of the risk-ratio. I^2^ and p values for heterogeneity are shown.

CI: confidence interval. EVD: external ventricular drain. IVF: intra ventricular fibrinolysis. IVH: intra ventricular hemorrhage. RE: random effects.

**Supplementary Figure 5**

**
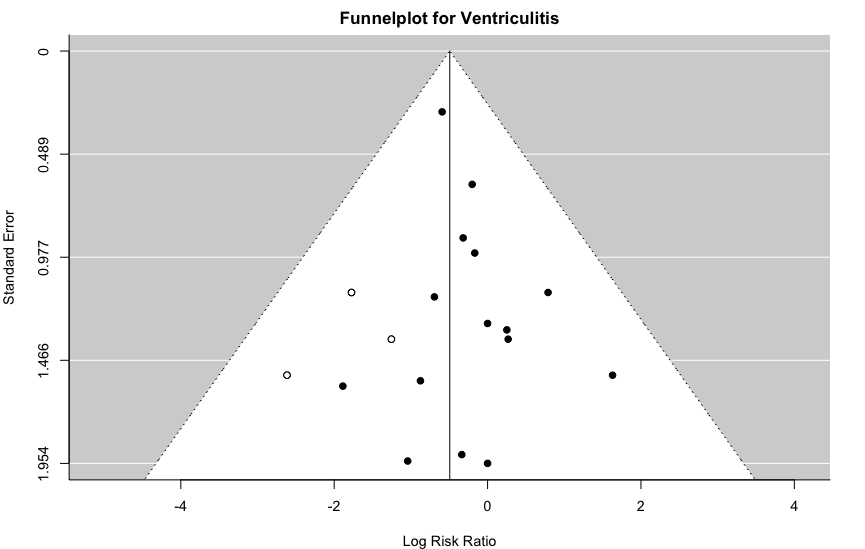
**

**Supplementary Figure 5:** Funnel plot for ventriculitis, after Trim and Fill.

• included studies. ° ‘missed’ studies indicated by Trim and Fill method.

**Supplementary Figure 6**

**
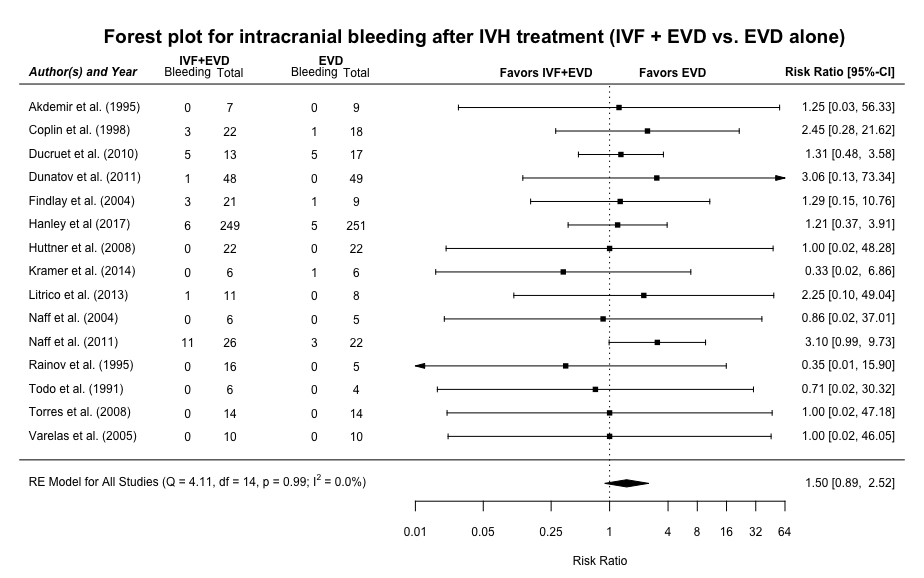
**

**Supplementary Figure 6:** Forest Plot for Intracerebral Bleeding after Intraventricular Hemorrhage.

Forest plot shows the pooled risk ratio for an intracerebral bleeding after start of treatment in patients receiving IVF and EVD, and those being treated with EVD alone using a random-effects model. Solid squares represent the point estimate of each study, with 95%-CI being shown in error bars. Thex diamond represents the pooled estimate of the risk-ratio. I^2^ and p values for heterogeneity are shown.

CI: confidence interval. EVD: external ventricular drain. IVF: intra ventricular fibrinolysis. IVH: intra ventricular hemorrhage. RE: random effects.

**Supplementary Figure 7
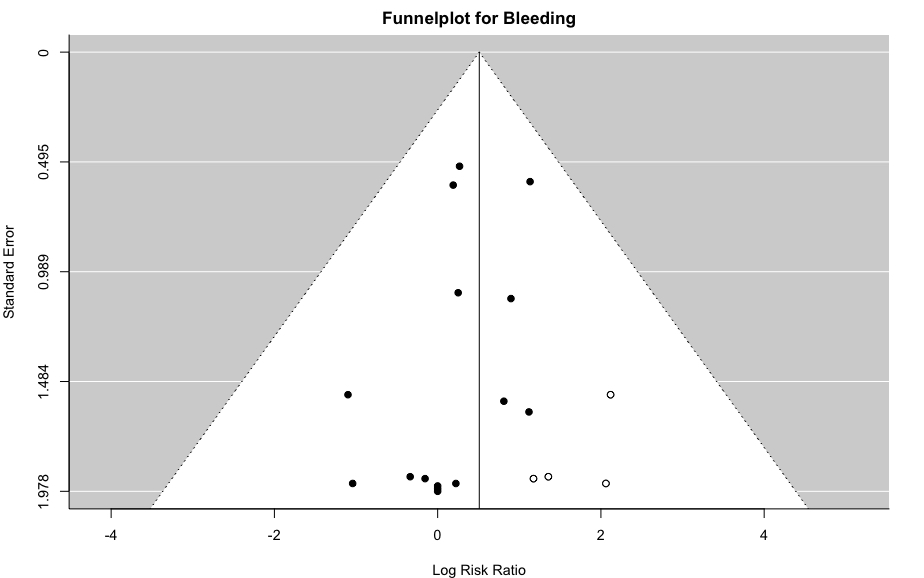
**

**Supplementary Figure 7:** Funnel plot for intracerebral bleeding, after Trim and Fill.

• included studies. ° ‘missed’ studies indicated by Trim and Fill method.

**Supplementary Figure 8**

**
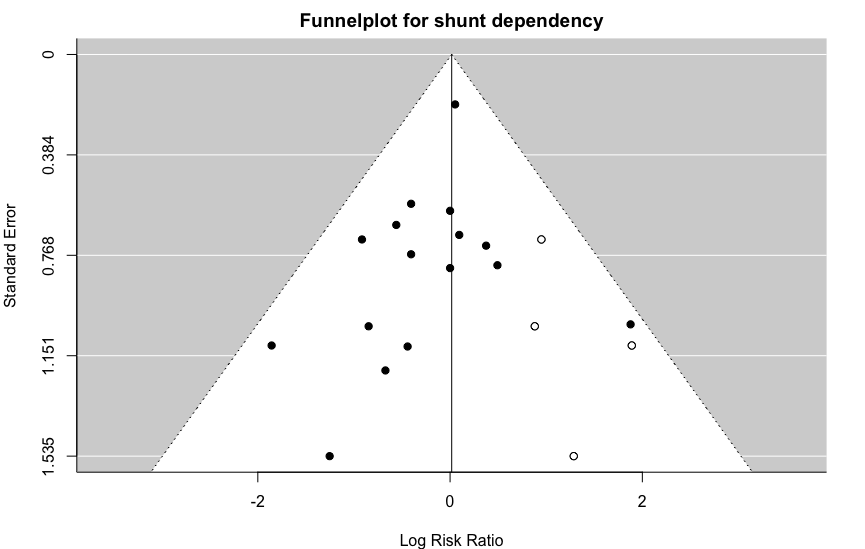
**

**Supplementary Figure 8:** Funnel plot for shunt dependency, after Trim and Fill.

• included studies. ° ‘missed’ studies indicated by Trim and Fill method.
